# Incidence Proportion and Clinicopathological Characteristics of Phyllodes Tumors of the Breast Among Patients With Breast Lumps in Dubai, 2017-2022: A Retrospective Observational Study

**DOI:** 10.7759/cureus.113942

**Published:** 2026-08-04

**Authors:** Omar Alsayed, Warda Siddiqi, Amir Saber

**Affiliations:** 1 General Surgery, Division of Surgery, Dubai Health, Dubai, ARE; 2 College of Medicine, Mohammed Bin Rashid University of Medicine and Health Sciences, Dubai, ARE

**Keywords:** breast disease, breast lump(s), fibroepithelial neoplasms, incidence, phyllodes tumor

## Abstract

Background: Phyllodes tumors are rare fibroepithelial neoplasms of the breast, with histological behavior ranging from benign to malignant. Published data from the Middle East are limited.

Objective: This study aimed to determine the incidence proportion and clinicopathological characteristics of phyllodes tumors among patients presenting with breast lumps at a tertiary care center in Dubai between 2017 and 2022.

Methods: This retrospective observational study included all female patients who presented with a breast lump to Dubai Hospital between January 2017 and December 2022 and underwent biopsy or surgical excision, with histopathology results available. The source population comprised 1,680 breast lump cases. Histologically confirmed phyllodes tumors constituted the analytic cohort. Variables analyzed included age, body mass index (BMI), tumor size, WHO histological subtype, margin status, surgical management, and 24-hour local recurrence. The incidence proportion was calculated with 95% confidence intervals using the Wilson score method. Fisher’s exact tests were used to explore associations between clinicopathological factors and recurrence; all tests used a prespecified significance threshold of α = 0.05.

Results: Twenty-four phyllodes tumors were identified among 1,680 breast lump presentations (incidence proportion: 1.43%; 95% CI: 0.89-2.10%). WHO subtypes were equally distributed: benign (n = 8, 33.3%), borderline (n = 8, 33.3%), and malignant (n = 8, 33.3%). The mean age was 41.7 years (SD 11.1), and the mean tumor size was 11.1 cm (SD 9.9). Negative surgical margins were achieved in 11/24 cases (45.8%). Preoperative core biopsy confirmed phyllodes tumors in only 5/24 patients (20.8%). Local recurrence within 24 months occurred in 2/24 patients (8.3%; 95% CI: 1.0-26.0%), both of whom had borderline or malignant tumors.

Conclusion: Phyllodes tumors comprised 24/1,680 (1.43%) of surgically evaluated breast lumps at this tertiary care center in Dubai, an estimate exceeding the commonly cited global reference range of 0.3-1%, although direct comparison is limited by differences in denominator definition. These findings represent the first incidence proportion data for phyllodes tumors from the Dubai region and highlight diagnostic challenges, an atypical subtype distribution, and a low short-term recurrence rate, collectively informing risk-adapted surgical management in a diverse Middle Eastern population.

## Introduction

Phyllodes tumors are rare fibroepithelial lesions of the breast that arise from the intralobular stroma and show a biphasic proliferation of stromal and epithelial components [1,2]. They account for approximately 0.3-1% of all primary breast tumors and around 2-3% of fibroepithelial breast tumors, underscoring their rarity in standard clinical breast evaluation [1,2]. Zhang and Kleer describe phyllodes tumors as nonductal lesions that originate in the connective tissue of the breast and can occur across a wide age range [1].

Histologically, phyllodes tumors are classified by the World Health Organization (WHO) as benign, borderline, or malignant based on stromal cellularity, nuclear atypia, mitotic activity, stromal overgrowth, and the nature of tumor margins [2-4]. A multidisciplinary consensus review by the pathologists involved in developing this grading scheme emphasized that the reproducibility of these criteria remains imperfect, even among experienced observers, particularly for borderline lesions [3]. Most published cohorts report that benign tumors comprise roughly 60% of cases, borderline tumors about 20%, and malignant tumors another 20%, although distributions vary by cohort and referral pattern [2-4]. While many phyllodes tumors behave in a relatively indolent fashion, malignant lesions carry a higher risk of local recurrence and distant metastasis, and even benign tumors have an inherent potential for local relapse [2-5].

Several lines of evidence inform current surgical management. A systematic review and meta-analysis pooling over 9,000 cases found that benign and borderline phyllodes tumors have relatively low recurrence rates even with narrower margins, while high-risk features such as large tumor size, stromal overgrowth, and malignant histology are more strongly linked to relapse [2,5,6]. A large nomogram-based cohort study similarly identified histological grade, more than margin width, as the dominant independent predictor of recurrence-free survival, consistent with an earlier analysis by Kim et al., which reported histological grade as the principal determinant of recurrence, with margin involvement as an additional contributing factor [5,7]. Nonetheless, the optimal margin width and its overall impact on recurrence, particularly for benign and borderline tumors, remain subjects of ongoing debate [8]. Reflecting this uncertainty, surgical excision remains the standard of care for phyllodes tumors, with the traditional goal of achieving negative margins to reduce local recurrence [9,10].

Clinically and radiologically, phyllodes tumors often resemble fibroadenomas, and distinguishing between these entities, especially on core needle biopsy, can be challenging [2-4]. This overlap can delay definitive diagnosis or lead to re-excision when a lesion initially presumed to be a fibroadenoma is reclassified as a phyllodes tumor on excision histology [2-4]. Accurate identification and risk stratification are therefore essential to optimize surgical planning and follow-up [4,9].

Despite multiple institutional reports from Europe, Asia, and the Americas, published data from the Middle East on the incidence, histological spectrum, and outcomes of phyllodes tumors remain limited [9,10]. In particular, there are few data describing the incidence proportion of phyllodes tumors among all patients presenting with breast lumps in the Gulf region or the relationship between margin status and recurrence in this setting [9,10]. Dubai Hospital, a large tertiary care center serving a diverse population, provides an opportunity to characterize the burden and behavior of phyllodes tumors in the United Arab Emirates.

This retrospective study examined all female patients presenting with breast lumps to Dubai Hospital between January 2017 and December 2022 to quantify the incidence proportion of histologically confirmed phyllodes tumors in this population. By examining tumor size, WHO subtype, margin status, and recurrence patterns alongside patient demographics, this study seeks to address the regional evidence gap and inform local clinical practice [9,10]. This study is reported in accordance with the STROBE (Strengthening the Reporting of Observational Studies in Epidemiology) statement for observational studies [11].

Aims and objectives

The primary aim was to determine the incidence proportion of phyllodes tumors of the breast among patients presenting with breast lumps at Dubai Hospital between January 2017 and December 2022. The secondary objectives were to calculate the incidence proportion of histologically confirmed phyllodes tumors among all patients with breast lumps who underwent surgical excision or biopsy and had available histopathology results; to describe the distribution of WHO histological subtypes (benign, borderline, and malignant) among the phyllodes tumors identified in this cohort; to evaluate modes of diagnosis, including Breast Imaging Reporting and Data System (BI-RADS) classification and core needle biopsy accuracy, as well as surgical management outcomes, including negative-margin rates and re-excision patterns; to estimate local recurrence rates within 24 months, overall and stratified by histological subtype, margin status, and re-excision status; and to characterize the demographic features (age, nationality, and BMI) and clinical characteristics (tumor size) of patients diagnosed with phyllodes tumors in this population.

## Materials and methods

Study design and setting

This retrospective observational study was conducted at Dubai Hospital, a tertiary care center in Dubai, United Arab Emirates, and is reported in accordance with the STROBE statement for observational studies [11]. Clinical, pathological, and outcome data were retrieved from the hospital electronic medical record (EMR) system for the period from January 1, 2017, to December 31, 2022. Follow-up data for recurrence ascertainment were collected through December 2024, providing a minimum potential follow-up of 24 months for all included cases.

Study population, denominator, and analytic cohort

The source population comprised all patients assigned female at birth who presented with a new breast lump or mass to Dubai Hospital during the study period and underwent biopsy or surgical excision, with histopathology results available in the EMR. The source population was identified through a structured EMR query using diagnostic and procedural codes for breast lumps, breast masses, fibroepithelial lesions, and core needle or excisional breast biopsies. Aggregate counts confirmed that 1,680 patients met the source population criteria over the six-year period; all had at least one histopathological result recorded in the EMR. This population served as the denominator for incidence proportion calculations.

From the source population, the analytic cohort comprised all patients with a histopathology report confirming a diagnosis of phyllodes tumor, classified by a consultant pathologist according to the WHO 2022 criteria. Patients whose definitive surgical treatment was performed exclusively at another institution, those with incomplete histopathological reports, those with insufficient clinical data to confirm the primary diagnosis, and those with a documented prior diagnosis of phyllodes tumor at Dubai Hospital or elsewhere were excluded; the latter exclusion ensured that all included cases represented primary rather than recurrent tumors.

Inclusion criteria

Female patients (assigned female at birth) of any age or nationality who presented with a new breast lump or mass to Dubai Hospital between January 1, 2017, and December 31, 2022; underwent biopsy or surgical excision (core needle biopsy, vacuum-assisted biopsy, lumpectomy, wide local excision, or mastectomy); had histopathology results available in the EMR; and had no prior diagnosis of phyllodes tumor were included.

Exclusion criteria

Patients whose index breast surgery or definitive treatment was performed exclusively at another hospital; patients with missing or incomplete histopathology reports for the index lesion; patients lacking sufficient clinical data to confirm the diagnosis or outcomes; and patients with a documented prior diagnosis of phyllodes tumor (to exclude recurrent cases from the primary incidence calculation) were excluded.

Data sources and variables

Data were extracted using a standardized collection form from medical charts, operative notes, radiology reports, and pathology reports. Variables included age at diagnosis; body mass index (BMI); nationality; tumor laterality; presenting symptom (palpable lump, pain, or rapid enlargement); imaging findings, including the BI-RADS category on ultrasound and mammography, where performed; preoperative core needle biopsy result; WHO histological subtype (benign, borderline, or malignant); maximum tumor diameter; margin status (negative: no tumor on ink; positive: tumor at or within the institutional margin threshold); surgical modality (wide local excision or mastectomy); re-excision status (performed, not performed, or not applicable, with the latter defined as patients who underwent mastectomy or achieved unambiguously clear margins at index surgery); and local recurrence within 24 months.

Recurrence data were ascertained from clinical follow-up notes, surveillance imaging reports, and repeat pathology results. Local recurrence was defined as a histologically confirmed phyllodes tumor in the ipsilateral breast following a disease-free interval after index surgery. Patients who did not attend follow-up within the 24-month window were censored at the last documented clinical contact. The aggregate denominator population count (n = 1,680) was verified through a secondary hospital records query.

Variable definitions

Phyllodes tumors were defined as fibroepithelial breast tumors diagnosed by a consultant pathologist based on established histological criteria, including stromal overgrowth, leaf-like architecture, and characteristic stromal morphology. WHO classification into benign, borderline, and malignant subtypes followed published criteria incorporating stromal cellularity, atypia, mitotic activity, stromal overgrowth, and margin appearance. The incidence proportion was calculated as the number of histologically confirmed phyllodes tumors divided by the total number of patients with breast lumps and available histology results during the study period, expressed as a percentage.

Data management

All extracted data were entered into a password-protected electronic database stored on a secure institutional server. Only de-identified study IDs were used in the analytic dataset; no names, medical record numbers, or other directly identifiable information were included in exported files. Access was restricted to study investigators via individual login credentials. Data quality checks included verification of outliers, range checks for continuous variables, and cross-validation of key variables (histological subtype and margin status) against original pathology reports.

Statistical analysis

Continuous variables were assessed for normality using the Shapiro-Wilk test. Age and BMI, which were normally distributed, were summarized as mean ± standard deviation (SD). Tumor size, which was non-normally distributed (right-skewed), was summarized as the median with interquartile range (IQR), with the mean and SD also reported for descriptive comparability. Categorical variables were summarized as counts and percentages, reported throughout as n (%).

The incidence proportion of phyllodes tumors among breast lump presentations was calculated as the number of confirmed cases divided by the total source population (n = 1,680) and expressed as a percentage, with 95% confidence intervals computed using the Wilson score method.

For contextual comparison with the widely cited international reference range of 0.3-1% of all primary breast tumors, a two-sided one-sample z-test was applied. This comparison is explicitly exploratory: the international reference estimate uses all histologically confirmed breast tumors as its denominator, whereas our denominator encompasses all breast lump clinic presentations regardless of final diagnosis, representing a broader and non-equivalent reference population. The results of this z-test should be interpreted in light of this denominator discrepancy.

Continuous variables were compared across the three WHO subtype groups using one-way analysis of variance (ANOVA), with the F statistic reported for normally distributed variables (age and BMI), and the Kruskal-Wallis test, with the H statistic reported for the non-normally distributed variable (tumor size). Associations between clinicopathological factors (WHO subtype, margin status, and re-excision status) and 24-month recurrence were evaluated using Fisher’s exact test or the Freeman-Halton extension for variables with more than two categories. Because expected cell counts were below 5 in more than 20% of cells for these small subgroup comparisons, exact tests were prespecified as the primary inferential tests, with the corresponding Pearson chi-square (χ²) statistic reported alongside for reference. A prespecified significance threshold of α = 0.05 was applied to all tests. Given the small analytic cohort (n = 24) and multiple comparisons across exploratory subgroups, all inferential results should be considered hypothesis-generating; no correction for multiple testing was applied, and the findings should not be used to modify established clinical guidelines for individual patients. All analyses were performed using IBM SPSS Statistics for Windows, Version 28 (Released 2021; IBM Corp., Armonk, New York).

## Results

Patient and tumor characteristics

Twenty-four patients with histologically confirmed phyllodes tumors were included in the analytic cohort. The mean age at diagnosis was 41.7 years (SD 11.1; range 17-56), with a median age of 44.5 years. The mean body mass index (BMI) was 27.6 kg/m² (SD 6.1; range 17.5-40.7). Tumors were large overall, with a mean maximum diameter of 11.1 cm (SD 9.9; range 1-35) and a median maximum diameter of 5.7 cm (IQR 3.0-15.5), reflecting the often substantial size at presentation described in the literature.

The cohort comprised a multinational population reflecting Dubai’s diverse demographics: 9 Emirati patients (37.5%), 11 Filipino patients (45.8%), and one patient each from Nigeria, Saudi Arabia, Sri Lanka, and Canada (4.2% each). This distribution mirrors Dubai Health Authority registrant demographics and is presented as a descriptive characteristic to support generalizability across Gulf providers; ethnicity-specific risk inference was not possible in this small cohort (Table 1).

**Table 1:** Patient and Tumor Characteristics of Histologically Confirmed Phyllodes Tumors (n = 24), Dubai Hospital, 2017–2022 Age and BMI are approximately normally distributed (Shapiro-Wilk p>0.05) and are presented as mean ± SD. Tumor size is right-skewed (Shapiro-Wilk p = 0.02) and is presented as median (IQR) with mean ± SD for descriptive comparability. 95% CI for recurrence calculated by the Wilson score method. SD: standard deviation; IQR: interquartile range; BI-RADS: Breast Imaging Reporting and Data System; WHO: World Health Organization; WLE: wide local excision; CI: confidence interval.

Variable	n (%) or Mean ± SD / Median (IQR)
Demographics	
Age at diagnosis (years)	
Mean ± SD	41.7 ± 11.1
Median (IQR)	44.5 (33.5–52.0)
Range	17–56
Body mass index (kg/m²)	
Mean ± SD	27.6 ± 6.1
Range	17.5–40.7
Nationality	
Emirati	9 (37.5%)
Filipino	11 (45.8%)
Other (Nigerian, Saudi, Sri Lankan, Canadian; n = 1 each)	4 (16.7%)
Clinical Presentation	
Side of tumor	
Right breast	12 (50.0%)
Left breast	12 (50.0%)
Presenting symptom	
Palpable lump	22 (91.7%)
Pain/discomfort	1 (4.2%)
Rapid enlargement of existing mass	1 (4.2%)
Pre-operative Imaging	
Ultrasound performed	24 (100%)
Ultrasound BI-RADS category	
BI-RADS 2 (benign)	4 (16.7%)
BI-RADS 4 (suspicious)	14 (58.3%)
BI-RADS 5 (highly suspicious)	4 (16.7%)
BI-RADS 6 (biopsy-proven malignancy)	1 (4.2%)
No assessment (code 0)	1 (4.2%)
Mammography performed	7 (29.2%)
Mammography BI-RADS category (among 7 imaged)	
BI-RADS 0 (incomplete)	2 (28.6%)
BI-RADS 3 (probably benign)	2 (28.6%)
BI-RADS 4 (suspicious)	3 (42.9%)
Pre-operative Core Needle Biopsy	
Core biopsy performed	19 (79.2%)
Core biopsy not performed	5 (20.8%)
Core biopsy result (among 19 biopsied)	
Phyllodes tumor (confirmed)	5 (26.3% of biopsied; 20.8% of analytic cohort)
Non-specific fibroepithelial lesion	10 (52.6% of biopsied; 41.7% of analytic cohort)
Spindle cell lesion	4 (21.1% of biopsied; 16.7% of analytic cohort)
Tumor Pathology	
Maximum tumor diameter (cm)	
Mean ± SD	11.1 ± 9.9
Median (IQR)	5.7 (3.0–15.5)
Range	1–35
WHO histological subtype	
Benign	8 (33.3%)
Borderline	8 (33.3%)
Malignant	8 (33.3%)
Surgical Management and Margin Status	
Surgical modality	
Wide local excision (WLE)	20 (83.3%)
Mastectomy	4 (16.7%)
Margin status	
Negative (no tumor on ink)	11 (45.8%)
Positive (tumor at or within margin)	13 (54.2%)
Re-excision status	
Not applicable (mastectomy or clear margins)	12 (50.0%)
Re-excision performed	2 (8.3%)
Re-excision not performed (positive margins, declined/lost)	10 (41.7%)
24-Month Local Recurrence	
Recurrence-free at 24 months	22 (91.7%)
Local recurrence within 24 months	2 (8.3%); 95% CI 1.0–26.0%
Median time to recurrence (months)	11 (range 9–13)

Imaging and preoperative histology

Ultrasound was performed in all patients. Most lesions (14/24; 58.3%) were categorized as BI-RADS 4, with four patients (16.7%) reported as BI-RADS 2, four (16.7%) as BI-RADS 5, one (4.2%) as BI-RADS 6, and one case (4.2%) with no ultrasound assessment recorded. Mammography was less frequently performed: 17/24 patients (70.8%) had no mammogram or it was deemed not applicable, while among imaged patients, three (12.5%) were BI-RADS 4, two (8.3%) were BI-RADS 3, and two (8.3%) were BI-RADS 0. This pattern is consistent with the reported reliance on ultrasound in younger and mixed-age cohorts and the frequent classification of phyllodes tumors as suspicious (BI-RADS 4-5) lesions.

Core needle biopsy was not performed in 5/24 patients (20.8%). Among the 19 patients who underwent core biopsy, histology was coded as phyllodes in 5 (20.8% of the analytic cohort), fibroepithelial lesion in 10 (41.7%), and spindle cell lesion in 4 (16.7%). Thus, only one in five patients had a definitive preoperative diagnosis of phyllodes, whereas the majority were reported as nonspecific fibroepithelial or spindle cell lesions, underscoring the well-recognized difficulty in distinguishing phyllodes tumors from fibroadenomas and other fibroepithelial lesions on core samples.

Histological subtype

All three WHO phyllodes subtypes were evenly represented: benign tumors accounted for 8/24 cases (33.3%), borderline tumors for 8/24 (33.3%), and malignant tumors for 8/24 (33.3%). This equal distribution differs from published cohorts, which typically report a predominance of benign tumors (50-70%); the implications of this unusual distribution are discussed below (Table 2).

**Table 2:** Clinicopathological Characteristics Stratified by WHO Histological Subtype (n = 24) ᵃ One-way ANOVA; F statistic calculated from group means, standard deviations, and sample sizes (df between = 2, df within = 21). ᵇ Kruskal-Wallis test. The exact H statistic requires the original ranked tumor-size values by WHO subtype and could not be recalculated from the summary median/IQR figures reported here. The overall-sample descriptive output (explore procedure) reviewed for this revision did not include a subtype-stratified Kruskal-Wallis analysis; the p-value (0.42) is retained from the original SPSS output. The corresponding H value should be inserted from a K-independent samples (Kruskal-Wallis) test with tumor subtype as the grouping variable once available. ᶜ Pearson chi-square (χ²) statistic shown for reference alongside the exact test. Because more than 20% of expected cell counts were below 5 in these 3 × 2 tables, Fisher's exact test (2-category rows) or the Freeman-Halton extension (three-category rows) was pre-specified as the primary inferential statistic; the reported p-values correspond to these exact tests, not to the χ² approximation. ᵈ Value confirmed against original SPSS CROSSTABS output (Pearson χ²(2) = 1.091, exact p = 1.000). Pre-specified significance threshold α = 0.05. All comparisons are exploratory, given the small analytic cohort (n = 24); findings should be considered hypothesis-generating. No correction for multiple testing was applied. SD: standard deviation; IQR: interquartile range; BMI: body mass index; BI-RADS: Breast Imaging Reporting and Data System; CI: confidence interval; WHO: World Health Organization.

Characteristics	Benign (n = 8)	Borderline (n = 8)	Malignant (n = 8)	Test Statistics	p-value
Demographics					
Age (years), mean ± SD	43.1 ± 12.4	42.5 ± 11.8	39.5 ± 9.8	F(2,21) = 0.23ᵃ	0.81
BMI (kg/m²), mean ± SD	26.8 ± 5.9	28.1 ± 6.5	27.9 ± 6.2	F(2,21) = 0.10ᵃ	0.89
Tumor Characteristics					
Tumor size (cm), median (IQR)	4.5 (2.5–8.0)	6.0 (3.5–15.0)	7.5 (4.0–22.0)	H(2) = n/aᵇ	0.42
Side: right, n (%)	4 (50.0%)	4 (50.0%)	4 (50.0%)	χ²(2) = 0.00ᶜ	1.00
Pre-operative Diagnosis					
Core biopsy performed, n (%)	7 (87.5%)	6 (75.0%)	6 (75.0%)	χ²(2) = 0.51ᶜ	0.84
Pre-op phyllodes confirmed, n (%)	1 (12.5%)	2 (25.0%)	2 (25.0%)	χ²(2) = 0.51ᶜ	0.81
BI-RADS 4-5 on ultrasound, n (%)	5 (62.5%)	7 (87.5%)	6 (75.0%)	χ²(2) = 1.33ᶜ	0.56
Surgical Management					
Mastectomy, n (%)	1 (12.5%)	1 (12.5%)	2 (25.0%)	χ²(2) = 0.60ᶜ	0.75
Negative margins achieved, n (%)	5 (62.5%)	4 (50.0%)	2 (25.0%)	χ²(2) = 2.35ᶜ	0.24
Re-excision performed, n (%)	0 (0.0%)	1 (12.5%)	1 (12.5%)	χ²(2) = 1.09ᶜ	0.61
24-Month Local Recurrence					
Recurrence, n (%)	0 (0.0%)	1 (12.5%)	1 (12.5%)	χ²(2) = 1.091ᶜ^,^ᵈ	1.000ᵈ
95% CI for recurrence	0-36.9%	0.3-52.7%	0.3-52.7%	—	—

Surgical margins and re-excision

Margin status was documented for all patients. Negative margins were achieved in 11/24 (45.8%), whereas 13/24 (54.2%) had positive margins. Re-excision status was coded as not applicable in 12/24 (50.0%), encompassing patients who underwent mastectomy or had unambiguously clear margins at index surgery; performed in 2/24 (8.3%); and not done in 10/24 (41.7%), representing patients with positive margins who did not subsequently undergo re-excision.

In the cross-tabulation of margin status versus 24-month recurrence, no recurrences occurred in the negative-margin group (0/11; 0.0%; 95% CI: 0-28.5%), while two recurrences were observed among patients with positive margins (2/13; 15.4%; 95% CI: 1.9-45.4%). The association between margin status and recurrence did not reach statistical significance (uncorrected χ²(1) = 1.846; Yates-corrected χ²(1) = 0.381; Fisher’s exact test, two-sided p = 0.482), reflecting the limited statistical power of this small cohort rather than confirming equivalence between the groups. Fisher’s exact test, rather than the χ² approximation, was used as the primary inferential test because expected cell counts were below 5 in this 2 × 2 table (minimum expected count: 0.92).

Re-excision analysis showed a directionally consistent pattern: no recurrences occurred in the not-applicable group (0/12; 0.0%), one recurrence occurred among the two patients who underwent re-excision (1/2; 50.0%), and one occurred among the 10 patients for whom re-excision was not done despite positive margins (1/10; 10.0%). The Fisher-Freeman-Halton exact test for this three-level variable yielded χ²(2) = 5.673, p = 0.076, not reaching the prespecified α = 0.05 threshold in this sample.

Recurrence within 24 months

Two of 24 patients (8.3%; 95% CI: 1.0-26.0%) developed local recurrence within 24 months, while 22/24 (91.7%) remained recurrence-free. When stratified by histological subtype, no recurrences occurred among benign phyllodes tumors (0/8; 0.0%), while borderline and malignant tumors each had one recurrence among eight patients (12.5% each; 95% CI: 0.3-52.7%). The association between subtype and recurrence was not statistically significant (χ²(2) = 1.091; Fisher-Freeman-Halton exact p = 1.000), reflecting the very small subgroup sizes (Table 3).

**Table 3:** Twenty-Four-Month Local Recurrence Stratified by Margin Status and WHO Histological Subtype (n = 24) All p-values calculated using Fisher's exact test (Panel A, 2×2) or the Freeman-Halton exact extension (Panel B, 3×2), pre-specified as the primary inferential statistic given expected cell counts below 5. Corresponding Pearson χ² statistics are reported alongside for reference; the Yates (continuity-corrected) χ² is additionally reported for Panel A, given its 2×2 structure. Pre-specified significance threshold α = 0.05. 95% confidence intervals for proportions calculated using the Wilson score method. Non-significant p-values for subtype comparisons reflect the very small event count (n = 2 total recurrences) and limited statistical power rather than true equivalence between groups. All findings are exploratory and hypothesis-generating. CI: confidence interval; WHO: World Health Organization; neg: negative margin; pos: positive margin.

Variable	No Recurrence (n = 22) (91.7%)	Recurrence (n = 2) (8.3%)	Total (n = 24)	Test Statistic	p-value (Fisher's Exact)
Panel A: Recurrence by Margin Status					
Negative margin (n = 11)	11 (100.0%)	0 (0.0%)	11		
Positive margin (n = 13)	11 (84.6%)	2 (15.4%)	13	χ²(1) = 1.846; Yates-corrected 0.381	0.482
95% CI for recurrence	—	1.9-45.4%	—		
Absolute risk difference (positive vs negative margin)	—	+15.4%	—		
Panel B: Recurrence by WHO Histological Subtype					
Benign (n = 8)	8 (100.0%)	0 (0.0%)	8		
Borderline (n = 8)	7 (87.5%)	1 (12.5%)	8		
Malignant (n = 8)	7 (87.5%)	1 (12.5%)	8	χ²(2) = 1.091	1.000 (Freeman-Halton)
95% CI, benign/borderline/malignant	—	0% (0-36.9%)/12.5% (0.3-52.7%)/12.5% (0.3-52.7%)	—		
Panel C: Cross-Tabulation: Recurrence by Margin Status and WHO Subtype					
Benign	5 (45.5%) neg/3 (23.1%) pos	0	8	Subtype recurrence rate:	0/8 (0.0%)
Borderline	4 (36.4%) neg/4 (30.8%) pos	1	8	Subtype recurrence rate:	1/8 (12.5%)
Malignant	2 (18.2%) neg/6 (46.2%) pos	1	8	Subtype recurrence rate:	1/8 (12.5%)
Total	11 (100%) neg/13 (100%) pos	2	24	Overall recurrence rate:	2/24 (8.3%)

## Discussion

This single-center retrospective study identified 24 histologically confirmed phyllodes tumors among 1,680 breast lump presentations at Dubai Hospital over six years, yielding an incidence proportion of 1.43% (95% CI: 0.89-2.10%). This estimate exceeded the commonly cited international reference of 0.3-1% of all primary breast tumors [1,2]; however, this comparison must be interpreted with caution because the denominators are not equivalent: our denominator encompasses all breast lump clinic presentations, whereas the reference figure is calculated using all histologically confirmed breast tumors. A two-sided one-sample z-test yielded z = 2.12, p = 0.034, which is statistically significant at α = 0.05; however, given the denominator mismatch, this result should be understood as a reflection of methodological noncomparability rather than evidence of a genuinely elevated regional burden. The higher observed proportion most likely reflects the referral profile of a tertiary institution, which concentrates complex surgical breast cases, combined with the inherently broader denominator used in our calculation.

The mean patient age of 41.7 years (range: 17-56) is consistent with the established epidemiology of phyllodes tumors, which typically present in premenopausal or perimenopausal women, approximately one to two decades earlier than invasive ductal carcinoma [2,4]. The mean tumor size of 11.1 cm (median: 5.7 cm) reflects the characteristic rapid growth of phyllodes tumors and mirrors reports from other institutional series describing bulky palpable masses at presentation [2,9]. The nationality composition, predominantly Filipino (n = 11, 45.8%) and Emirati (n = 9, 37.5%), reflects Dubai Health Authority registrant demographics and, while precluding ethnicity-specific risk inference in this small cohort, demonstrates the generalizability of the findings to the broader multinational Gulf population.

The younger age profile observed in this cohort should also be considered against the broader regional pattern of earlier breast disease presentation described across the Gulf Cooperation Council and wider Arab region, where the mean age at breast cancer diagnosis has been reported to be approximately a decade earlier than that in Western populations [12,13]. Although this literature pertains predominantly to invasive carcinoma rather than fibroepithelial neoplasms, it raises the possibility that demographic and reproductive factors specific to this population, rather than phyllodes-specific biology, contribute to the age distribution observed here, a hypothesis that would require regional fibroepithelial tumor-specific data to confirm.

Preoperative diagnostic performance mirrored the well-recognized limitations of core biopsy in phyllodes tumors. Ultrasound was performed in all patients and classified 18/24 lesions (75%) as suspicious (BI-RADS 4 or 5), consistent with the sonographic appearance of phyllodes tumors as well-circumscribed solid masses with internal heterogeneity [2,4]. Despite this, definitive preoperative confirmation of phyllodes tumors was achieved in only 5/24 cases (20.8%); the majority were reported as nonspecific fibroepithelial (10/24; 41.7%) or spindle cell lesions (4/24; 16.7%). This underdiagnosis rate is attributable to the sampling limitations of core biopsy, which may not capture the leaf-like architecture or stromal overgrowth required for WHO classification, a limitation also highlighted by the multidisciplinary pathology consensus review by Tan et al., who noted imperfect interobserver reproducibility of grading criteria even on full excision specimens, let alone limited core samples [2-4]. These findings reinforce the need for multidisciplinary radiology-pathology correlation when a large or rapidly enlarging fibroepithelial lesion is encountered, particularly when core biopsy returns a nonspecific result. These findings are consistent with published diagnostic accuracy data: core needle biopsy sensitivity for phyllodes tumors has been reported in the range of 45-83%, with specificity generally exceeding 90% but with a persistently high false-negative rate driven by sampling of the epithelial rather than the stromal component [14-16]. Efforts to improve preoperative discrimination have included combining ultrasound feature analysis with biopsy [14] and, more recently, shear-wave elastography, which may help flag stromal heterogeneity suggestive of phyllodes tumors in equivocal fibroepithelial lesions on core biopsy, although this modality remains investigational and was not available for review in the imaging performed in the present cohort [17]. Quantitatively, published series report interobserver agreement for distinguishing phyllodes tumors from cellular fibroadenomas in the fair-to-moderate range (κ approximately 0.5-0.66), reinforcing that the diagnostic uncertainty observed in our cohort reflects a recognized limitation of the diagnostic pathway rather than an institution-specific deficiency [18,19].

The equal distribution of benign, borderline, and malignant subtypes (n = 8 each, 33.3%) is atypical compared with most published cohorts, which report a benign predominance of 50-70%; for comparison, a 404-patient single-center cohort reported a subtype distribution of 41.6% benign, 45.5% borderline, and 12.9% malignant [2,4,7]. This balanced distribution warrants careful consideration. Three explanations are plausible, though none can be confirmed from the present data. First, referral bias toward a tertiary center may enrich the case mix with higher-grade, more complex tumors requiring specialist surgical management. Second, the COVID-19 pandemic, which overlapped with the 2020-2021 segment of the study period, may have delayed the presentation of lower-grade, slower-growing benign tumors, while patients with rapidly enlarging malignant tumors still sought care, a phenomenon observed in other cancer registries during this period [10]. Third, the small analytic cohort (n = 24) introduces substantial sampling variability, and a chance distribution cannot be excluded. At a biological level, the existence of three histologically distinct subtypes is increasingly understood to reflect divergent molecular pathogenesis rather than a single continuum of severity: MED12 mutations, present in a majority of fibroadenomas and benign phyllodes tumors, become progressively less frequent with increasing grade, while malignant phyllodes tumors accumulate additional driver mutations and greater genomic complexity [20]. This molecular heterogeneity may partly explain why subtype distribution varies so widely across published cohorts and supports the argument that the balanced distribution observed here, while unusual, is biologically plausible rather than necessarily an artifact of small sample size alone. Future multicenter Gulf region studies with larger case volumes will be necessary to determine whether this distribution reflects a genuine regional biological or epidemiological pattern.

Negative margins were achieved in 11/24 cases (45.8%), a rate lower than ideally desired but consistent with reports from settings where large tumor size at presentation limits the feasibility of wide excision without cosmetic or functional compromise [5,8,10]. Local recurrence within 24 months occurred in 2 of 24 patients (8.3%; 95% CI 1.0-26.0%), both in the borderline and malignant subtype groups (1/8 each, 12.5%), with no recurrences among the 8 benign cases. This overall 24-month recurrence rate lies within the lower-to-mid range reported in comparable institutional series of mixed-grade cohorts [6,7]. The subtype-specific recurrence gradient observed here is consistent with established evidence that histological grade is the principal determinant of recurrence risk: a systematic review and meta-analysis pooling over 9,000 cases reported subtype-specific local recurrence rates of approximately 8% for benign, 13% for borderline, and 19-30% for malignant tumors, and a separate 404-patient nomogram-based cohort reported comparable rates of 3.6%, 14.1%, and 42.3%, respectively [5-7]. Although our subtype-specific recurrence rates (0%, 12.5%, and 12.5%) are numerically lower than these pooled estimates, most plausibly because of our shorter 24-month follow-up window compared with the 5-10-year follow-up periods used in most published series rather than a genuinely more favorable disease course, the directional pattern of recurrence among the higher histological grades is concordant with this broader literature. Fisher’s exact test did not demonstrate a statistically significant association between margin status and recurrence (χ²(1) = 1.846, Yates-corrected χ²(1) = 0.381; p = 0.482) or between WHO subtype and recurrence (χ²(2) = 1.091; p = 1.000). These null results should not be interpreted as evidence that margin status and subtype are clinically unimportant; rather, they reflect the very limited statistical power of a 24-patient cohort. The absolute directional pattern, with both recurrences occurring in margin-positive, higher-grade cases, is consistent with the literature [5,6]. All subgroup comparisons in this study are exploratory and hypothesis-generating. The role of adjuvant radiotherapy in reducing local recurrence, particularly for borderline and malignant phyllodes tumors with positive or close margins, remains an area of active investigation that this study was not designed to address. Meta-analyses have reported pooled local recurrence rates of approximately 8% with adjuvant radiotherapy versus 12% with surgery alone [21], and a prospective multi-institutional cohort demonstrated a lower recurrence rate with margin-negative excision plus radiotherapy than that reported in historical series of margin-negative excision alone [22]. Single-institution retrospective data further suggest that this benefit may be most pronounced after breast-conserving surgery rather than mastectomy [23]. Given that both recurrences in the present cohort occurred in margin-positive, higher-grade tumors, these findings raise the question of whether adjuvant radiotherapy should have been considered for the affected patients; this was not systematically evaluated here and represents an important avenue for future prospective study in this population.

These data provide the first incidence proportion estimate for phyllodes tumors from the Dubai region, adding to a literature dominated by Western and East Asian cohorts [9,10]. The 1.43% incidence proportion among breast lump presentations establishes phyllodes tumors as rare but clinically relevant in Dubai. The balanced subtype distribution cautions against assuming benignancy in large or atypical fibroepithelial lesions and supports routine excision with margin assessment for cellular fibroepithelial tumors on core biopsy [2-4].

Limitations

This study has several limitations that require explicit acknowledgment. The retrospective single-center design and small analytic cohort (n = 24) substantially limit statistical power and generalizability; all inferential findings are exploratory. The study denominator comprises breast lump clinic presentations rather than all confirmed breast tumors, precluding direct comparison with international prevalence estimates and making the z-test comparison methodologically imperfect. The 24-month recurrence window is shorter than the 5-10-year follow-up recommended for phyllodes tumors, particularly malignant lesions, and likely underestimates cumulative recurrence. Overlap with the COVID-19 pandemic may have introduced ascertainment bias through disrupted follow-up and altered referral patterns in 2020-2021. Missing histopathological details, including mitotic count and stromal overgrowth data in some cases, precluded refined WHO risk stratification. Multiple comparisons across small subgroups increase the risk of type I error; all positive findings should be viewed as hypothesis-generating. Loss to follow-up may introduce attrition bias, and confounding by indication may affect associations involving margin re-excision, as higher-risk tumors likely prompted closer scrutiny.

Clinical implications and future directions

Pending prospective validation, these findings are consistent with a risk-adapted surgical approach: excision to negative margins for all phyllodes subtypes, selective re-excision for borderline and malignant tumors with focally positive margins, and short-interval surveillance imaging for higher-risk cases. For borderline and malignant tumors with persistently positive margins for which re-excision is not feasible, multidisciplinary discussion of adjuvant radiotherapy is reasonable, given the recurrence-reduction signal reported in the literature [21-23]. These recommendations should be understood as hypothesis-generating, given the small cohort size, and should not be used to override established institutional or guideline-based practice. Multicenter UAE and Gulf region registries with standardized pathological reporting and a minimum five-year follow-up would substantially advance the regional evidence base [9].

## Conclusions

This study provides the first regional estimate of the incidence proportion of phyllodes tumors in Dubai, identifying 24 cases (1.43%) among 1,680 breast lump presentations over six years. Relative to prior work, the cohort was distinguished by an unusually balanced WHO subtype distribution and tumors that were often large at diagnosis, while confirming that preoperative core biopsy frequently underclassifies these lesions as nonspecific fibroepithelial or spindle cell lesions. Recurrence within 24 months was infrequent and confined to borderline and malignant tumors, a pattern directionally consistent with grade-based recurrence models reported in larger cohorts. Because the analytic cohort was small, these associations should be treated as hypothesis-generating rather than definitive. The findings nonetheless offer a practical starting point for risk-adapted surgical planning in this population and underscore the need for larger, prospective, multi-institutional Gulf region studies with longer follow-up and standardized pathological reporting to confirm whether the observed subtype distribution and recurrence pattern reflect a genuine regional signal.
